# Current News and Opinion

**Published:** 1886-07

**Authors:** 


### Current News and Opinion.

### AMERICAN DENTAL ASSOCIATION.

The twenty-sixth annual meeting of the American Dental Association will be held at Niagara Falls, commencing at 10 A. M., on Tuesday, Aug. 3, 1886.

GEO. H. CUSHING, Secretary.

The Committee of Arrangements had hoped to give full and definite information in the July number of journals in regard to all the details of the arrangements for the annual meeting of the Association, to be held at Niagara, August 3d. But as the railroad rates thus far secured have not been as satisfactory as the committee yet hope to secure and are working for, they will issue a circular later to all members of the Association, and to local societies as far as possible. Those who are not members of the Association and who wish the circular, will please drop a postal to the Chairman of the Committee to insure their getting it. The railroad rates as thus far secured on all leading lines are one and a third fare round trip, to be issued upon presentation of certificate. Definite information concerning this will be given in the circular if better terms and arrangements are not secured.

The hotel rates will be as follows : The International Hotel will receive dentists and their families at $3,00 per day, the Cataract at $4.00 per day, the Niagara, Prospect Park and Hotel Atlantique $2.00 per day if rooms are applied for and secured in advance. The Park Theatre, adjoining the International Hotel, has been secured as a place ot meeting. Do not be anxious about not receiving the circular; you will get it some time in July, but a few days' delay in issuing the circular may mean a good deal of money saved to those attending the Association. For instance, the arrangements were not completed and circular issued until latter part of July last year. A month earlier it would have been impossible to have gotten the low rates finally secured. Remember, we promise nothing better than we now publish, but will continue to work for more favorable terms.

All State and local societies which have adopted substantially the Code of Ethics of the American Dental Association, will remember that they are entitled to one delegate for every five members. Such delegates must have credentials signed by the President and Secretary of the society which they represent.

J. N. CROUSE, Chairman Com. of Arr.,
2231 Prairie Ave., Chicago.

### NATIONAL ASSOCIATION OF DENTAL EXAMINERS.

The annual meeting will be held at 11 A. M., on Monday, Aug. 2, 1886, at Niagara Falls. It is hoped all State Boards will be represented.

GEO. H. CUSHING, Secretary.

### NOTES FROM THE WEEKLY MEDICAL REVIEW.

The *New York Medical Record* complains that the lines by Robert Burns :—"A chiel's amang you takin' notes, an' faith he'll prent it," are misquoted by the *Review*. We submit that a physician has a right to treat Burns as the exigencies of the case requires.

---

A gentleman taking advantage of the nice weather planted some seeds, among which he noticed some strange ones. As all the seeds came up except the strange ones, he dug a few of them up and submitted them to us. On examination they proved to be a gelatine-coated cathartic pill. He will have to wait awhile before the rest will bloom.—*National Druggist.*

We have frequently planted gelatine-coated cathartic pills in the stomachs of our victims, which manifested a much stronger inclination to "come up" than they did in the above instance.

---

It is officially announced that Senor Rafael Alcalde, of Brasil, has been appointed Surgeon-Dentist to the infant King of Spain, whose birth lately gave rise to so much rejoicing in the Spanish capital. The *Globe* inquires, very naturally, whether the young King was born with teeth. In any case, the part of this professor of the gentle dental art will, for some months to come, be as much a sinecure as that of the surgical instrument maker, who was recently addressed by a country customer as "suspensory bandage maker to Her Majesty the Queen."

---

*The Medical Press*, in the name of accuracy, protests against the allusion to Dr. Watson as the author of a "monogram" on Amputation.

We would remind our good friend from Buffalo that the "monogram" was the *initial essay* of Dr. Watson on the subject.

---

A carpenter knocked his rival's teeth out with an axe,and then claimed that it was an axe-dental operation.

---

### CHICAGO DENTAL CLUB.

A new dental society was organized in Chicago on May 18th, under the name of the Chicago Dental Club, and the following officers were elected :

*President*—L. P. Haskell, D. D. S.

*Vice-President*—Chas. P. Pruyn, M. D., D. D. S.

*Secretary*—Arthur B. Freeman, M. D., D. D. S.

*Treasurer*—Dr. E. M. S. Fernandez.

*Business Committee*—John S. Marshall, M. D.; E. S. Talbot, M. D., D. D. S., and Dr. I. A. Freeman.

This society will be in affiliation with the American Dental Association, and one of its chief aims will be the development of the younger men in the Chicago profession.

CHICAGO, June 1, 1886.

ARTHUR B. FREEMAN,
Recording Secretary.

### JURY DUTY.

In the INDEPENDENT PRACTITIONER for March, 1885, p. 169, is an item headed "Jury Duty," which states that according to chapter 358, Laws of New York, 1881, dentists are exempt from jury duty.

I have taken pains to look the matter up and find it erroneous. The impression is made that it applies to New York State.

The Code of Civil Procedure, under exemption of jurors, § 1030, subdivision 4, says: "A practicing physician or surgeon, having patients requiring his daily attention, is exempt from jury duty." This applies to New York State, *except* New York and Kings Counties.

§1081, subdivision 2, reads the same as chapter 358, Laws of New York, 1881, and applies to New York County only.

Chapter 358, Laws of New York, 1881, applies to Kings County only, as will be seen. It reads as follows: An act to amend the Code of Civil Procedure, passed May 25th, 1881. §1, Subdivision 2, of section eleven hundred and twenty-seven of the Code of Civil Procedure is hereby amended so as to read as follows:

2. A practicing physician, surgeon or dentist-surgeon, having patients requiring his daily professional attendance, and not following any other calling, and a licensed pharmaceutist or pharmacist, while actually engaged in his profession as a means of livelihood, shall be exempt from jury duty.

§2. This act shall take effect immediately.

It is thus seen that the general law, §1030, governs the exemptions outside of Kings County; §1081 those in New York County; and §1127, amended as chapter 358, Laws of New York, 1881, those of Kings County.

The special legislation for those two counties ought to be extended to the whole State, and I can only account for the exemption mentioned in the INDEPENDENT PRACTITIONER for March, 1885, p. 169, as being in Kings County, or that the judge who granted it did not take pains to see that that chapter referred exclusively to Kings County, through the §1127 amended.

W. C. HAYES, M. D. S., Albion, N. Y.

---

### DENTAL SOCIETY OF THE STATE OF NEW YORK.

At the annual meeting held at Albany, N. Y., May, 1886, the following officers were elected for the ensuing year:

*President*—Norman W. Kingsley, New York.

*Vice-President*—B. Rathbun, Dunkirk.

*Secretary*—J. Edw. Line, Rochester.

*Treasurer*—H. G. Mirick, Brooklyn.

*Correspondent*—W. H. Atkinson, New York.

A. M. Holmes, of Morrisville, and A. P. Southwick, of Buffalo, were re-elected members of the Board of Censors.

The degree of M. D. S. was conferred upon W. N. Frazer, of Danbury, Conn., J. L. Appleton, of Albany, N. Y., and J. H. Frull, Brooklyn, N. Y.

W. W. Walker, S. B. Bridge, O. J. Gross, W. C. Stewart, and F. A. Green were elected permanent members. J. EDW. LINE, Secretary.

### THRUSH IN CHILDREN.

Not long since I had brought to me a child of six months, suffering from the following symptoms :

Constipation, at times irregular action of bowles, regurgitation of food and an asthmatic cough. Its mouth was full of thrush sores, and its appearance one of poor nourishment.

It had been given a number of Infants' Foods in vain, one of which I prescribed myself.

By means of mild medication, directed towards the cough and stomach, something was accomplished. Finally I gave "CARNRICK'S SOLUBLE FOOD," and had the satisfaction of having it retained, and at last accounts the child was doing nicely.

I am inclined to think this food is worthy of attention on the part of the profession.

It recommends itself in that it contains caseine, rendered soluble by pancreatine, starch converted into dextrine and maltose. Hence it requires but little preparation, and that is so simple that mistakes cannot occur.

It requires no addition of milk.

It has the advantages and none of the disadvantages of the many foods now in the market, and forms a nearly physiological substitute for mother's milk.

Very truly, C. F. DENNY, in *Northwestern Lancet.*

ST. PAUL, June 1, 1886.

---

### A NEW HEMOSTATIC AGENT.

The following notice appears in the "*Popular Science News*" for June, 1886 ;

"Dr. Spaak, of Brussels, claims for the following simple solution excellent, not to say fabulous, results : Chloroform, two parts ; water, one hundred parts. He says that he has used this hemostatic liquid for several months, and attributes to it the following great advantages :

1st. It acts with truly wonderful rapidity ;

2d. It possesses no escharotic action ;

3d. It is to be had everywhere, and may be prepared instantaneously ;

4th. It costs very little ;

5th. It possesses no disagreeable effects, and does not hinder the surgeon in his operations.

At a recent meeting of the Louisville Medical Society, Surgeon Godfrey of the U. S. Marine Hospital Service detailed some interesting experiences with this mixture. In his opinion it is the coming hemostatic."

I have employed this preparation a number of times, and may add to the above that when used about the gums and teeth, it possesses for the Dentist the advantages of leaving no clot of blood nor discoloration of the surface.

I have used it upon cotton, and also applied it with a dental syringe or pipette, and have thus far found it satisfactory.

HENRY N. DODGE, M. D., D. D. S.,
MORRISTOWN, NEW JERSEY.

### THE INTERNATIONAL MEDICAL CONGRESS.

This journal has refrained from publishing the names of any of those who, it has been reported, have been appointed to the section of Oral and Dental Surgery in place of those who some time since resigned, and as additions to the Council. But Dr. A. E. Baldwin, of Chicago, sends to *The Journal of the British Dental Association* the following list, which appears in its May number :

*President*—J. Taft, Cincinnati, O.

*Secretaries*— { E. A. Bogue, New York, N. Y. / F. H. Rehwinkel, Chillicothe, O.

### COUNCIL.

| | |
|---|---|
| F. J. S. Gorgas, Baltimore, Md. | W. H. Morgan, Nashville, Tenn. |
| J. W. White, Philadelphia, Pa. | A. O. Hunt, Iowa City, Ia. |
| W. A. Spaulding, Minneapolis, Minn. | G. W. Keeley, Oxford, O. |
| C. A. Brackett, Newport, R. I. | J. McManus, Hartford, Conn. |
| J. Richardson, Terre Haute, Ind. | A. L. Northrop, New York, N. Y. |
| B. H. Catching, Atlanta, Ga. | W. C. Wardlaw, Augusta, Ga. |
| E. S. Chisholm, Tuscaloosa, Ala. | C. W. Spalding, St. Louis, Mo. |
| M. W. Foster, Baltimore, Md. | R. Finley Hunt, Washington, D. C. |
| C. F. W. Bodecker, New York, N. Y. | E. Palmer, La Crosse, Mo. |
| G. J. Friedrichs, New Orleans, La. | R. R. Andrews, Cambridge, Mass. |
| C. A. Marvin, Brooklyn, N. Y. | S. B. Palmer, Syracuse, N. Y. |

---

### ELECTRICITY AS AN ANÆSTHETIC.

History repeats itself. Circulars now haunt the profession, resurrecting electricity as a local anæsthetic. As far back as July, 1858, an article by "J. D. W.," on "The Galvanic Process" in extraction of teeth, appeared in the *Dental News Letter*, and in vol. xii. of the same journal, half a dozen more articles on extraction and extirpation of pulp, by electricity, were published. It was then the general craze. A lad, wishing to have several teeth extracted by electricity, once came to me, but when the forceps were presented to his mouth, he drew back and protested. He wanted them out by electricity, not with forceps.

After a thorough trial it was abandoned, as to many the electric shock was worse than simple extraction. With many patients it is the knowledge of the operations that kills, more than its severity, and such are not benefited by local anæsthetics. When the health, and even the life, of the patient seems to be in jeopardy, and the shock of extraction is more to be dreaded than the dangers of anæsthesia, it is best to give a general anæsthetic, because then we know that the operation can be performed without the fight or fright of consciousness. Electricity in such cases would be useless.

JNO. D. WINGATE,
Carbondale, Pa.

### DENTAL JOURNALS WANTED.

The editor of this journal will pay cash for the following numbers of dental journals, or an exchange will be made with those who desire to complete their own files.

THE DENTAL REGISTER.

Vol. III, Nos. 1, 2, 3.

" VI, " 1, 2, 3, 4.

" XXXVI, No. 1.

AMERICAN JOURNAL OF DENTAL SCIENCE (Third Series).

Vol. II, No. 8.
" V, " 3, 7, 11.
" VI, " 2, 3, 5, 7, 10.
" VII, " 2, 3, 7.
" VIII, " 6, 7, 10.

Dr. Frank Abbott, No. 22 West 40th Street, New York City desires the following numbers :

DENTAL REGISTER.

Vol. 12, No. 11.

AMERICAN JOURNAL OF DENTAL SCIENCE.

Vol. V, Nos. 3, 7.
" VI, " 2.
" XVI, " 9, 10, 11, 12.

---

### PENNSYLVANIA STATE DENTAL SOCIETY.

The 18th annual session of the Pennsylvania State Dental Society will be held at Cresson Springs, commencing Tuesday, July 27th, at 10 A. M., and continuing three days. Rates at the Mountain House, $3.00 per day, to delegates and their families. The Pennsylvania Central, Northern Central and Philadelphia and Erie Railroads will sell special excursion tickets, orders for which can be obtained from the Corresponding Secretary, otherwise regular excursion rates will be charged. Other roads will sell at the usual excursion rates. Programmes and general information to be obtained from

W. H. FUNDENBERG,
Corresponding Secretary,
958 Pennsylvania Ave., Pittsburg, Pa.

---

### MARRIED.

At Kansas City, Mo., Tuesday, May, 18th, by Rev. C. L. Thompson, Dr. J. D. Patterson, to Miss Carrie Cooper.

The many friends of Dr. Patterson will be glad to learn of what he has at last accomplished. We wish him and his estimable wife a long, happy, and prosperous life.

### SIXTH DISTRICT DENTAL SOCIETY.

The following is the list of officers elected at the seventeenth annual meeting of the Sixth District Dental Society of the State of New York:

*President*—G. W. Melotte, Ithaca.

*Vice-President*—S. W. Adamy, Union.

*Secretary*—E. D. Downs, Owego.

*Treasurer*—Frank B. Darby, Elmira.

*Censor*—C. G. Sumner, Norwich.

*Delegates to State Society*—G. W. Melotte, Ithaca, 4 years; Frank B. Darby, Elmira, 4 years; C E. Dunton, Cazenovia, 1 year.

---

### NEW JERSEY STATE DENTAL SOCIETY.

The sixteenth annual meeting of the New Jersey State Dental Society will be held at the Coleman House, Asbury Park, July 21, 22, and 23. Dr. Wm. Herbst, of Bremen, has kindly consented to give a clinic on his method of filling teeth by rotation. A cordial invitation is extended to all members of the profession to meet with us. Asbury Park is two hours from New York or Philadelphia, on the sea shore. Rates, $2.50 and $3 00 per day.

Chas. A. Meeker, D. D. S.,
Secretary.

---

### MINNESOTA.

The Minnesota State Dental Association will meet in the State Capitol at St. Paul, July 21st 22d and 23d. A cordial invitation is extended to members of the profession. M. G. Jenison, Secretary.

The Minnesota State Board of Dental Examiners will meet at St. Paul, in the Ryan Hotel, at 9 A. M., Friday, July 25th, immediately after the close of the Minnesota State Dental Society.

J. H. Martindale,
Secretary.

---

John Tomes, F. R. S., F. R. C. S., L D. S., of London, England, the well known dentist and writer upon dental subjects, and the Father of English Scientific Dentistry, has had the honor of knighthood conferred upon him by the Queen, and is now Sir John Tomes. If these decorations were always as worthily bestowed as in this instance, they would be valued even more highly than they now are.

---

The Annual Meeting of the British Dental Association will be held in London on the 19th, 20th and 21st of August, under the presidency of Sir Edwin Saunders. The Society is not strictly a scientific body, but it has under its charge the ethics and general management of professional affairs under the dental act.

The Secretary of "The Dentists' Scientific and Benevolent Association," which was organized about three years ago, sends out the announcement of the first assessment for the death of a member. Few dentists are acquainted with the Association, but it is a mutual assurance and benefit society formed in the west, and it would be well if every eastern dentist could belong to it. There is no reason why dentists, as well as members of other professions, should not have their benefit associations. Each of us can recall more than one case in which a dentist of prominence has died leaving little provision for his family. Had he been a member of such an organization as this, a fund, sufficient for immediate wants at least, would have been secured. Those who wish to know more of the Dentists' Benevolent Association can obtain the information by addressing the Secretary, Dr. R. I. Pearson, Kansas City, Mo.

---

In Manchester, England, recently, a dentist named Jackson was sued for civil damages for the alleged seduction and consequent pregnancy of a young woman aged twenty-five. It appears that she went to Jackson for the purpose of having a tooth extracted, and while under the influence of the gas she claimed that the defendant ravished her. The jury failed to agree. The case presents some unusual particulars. In the first place, the young woman made no complaint for a long time, and even permitted the banns of marriage between herself and a young man to whom she was engaged to be married to be published, without informing him of her condition. Then she preferred a civil claim for damages instead of making a criminal complaint. Every dentist knows that such a charge is grossly improbable, and the suit should properly have been dismissed.

---

Mr. J. W. Lambert, of "Listerine" fame, won great distinction at the late meeting of the American Medical Association. To him is given the credit for the local success of the meeting. When everything promised failure he organized victory, and by his personal efforts alone insured the proper entertainment and care of the guests, all the time modestly keeping himself in the background, never seeking notoriety, but quietly doing the work while others bore the honors. In recognition of his labors, and in gratitude to him, the committee of arrangements presented him with a beautiful Jurgensen watch, suitably engraved. "Listerine" must be a wonderful antiseptic. It saved the American Medical Association.

---

Dr. W. B. Slater, in *The Lancet*, describes the case of a working girl who had delirium tremens caused by chewing tea leaves. She had acquired the habit while working in a factory, and when she was deprived of the tea she was in a condition analogous to that of a confirmed inebriate when deprived of alcohol. The delirium tremens was well marked and characteristic.

---

Dr. Curran, in the *Southern California Practitioner*, says that some of his townsmen have lately gone to the City of Los Angeles for treatment, and "returned home with yards of tapeworm in bottles, very handsome, and doubtless worth all they cost." They can be kept as a lasting souvenir of the place, as they will not decay, *being made of celluloid.*

A Chicago Paper tells the following of the well-known Dr. J. Adams Allan, when he was commencing practice. One winter's day, all muffled, he was riding in a street car, when he overheard two persons talking about him. One asked the other what sort of a doctor was this Allan? "All I know of him is that he snatched my aunt from the grave last summer." "Did he, indeed," said the other, "well, then, he must be a pretty good doctor. What was the matter with your aunt?" "Oh! she was dead and buried, you know."

---

Goodyear Dental Vulcanite Company Redivivus.—A circular from a former agent informs those against whom decrees and damages were granted in favor of the company, and those who settled by notes not yet paid, that the interests of Josiah Bacon are now in the hands of the said agent, who is ready to receive the amounts due.

Josiah Bacon's body lies mouldering in the grave,
But his soul is marching on.

---

It is Estimated that coca is used by 10,000,000 of the human race; betel nut by 100,000,000; chickory by 40,000,000; coffee by 100,000.000; hashish is eaten or smoked by 300,000,000; opium by 400,000,000; 500,000,000 use tea, and all the known people of the earth use tobacco. The desire for some drug that has a specific action in the modification of nervous force seems universal, and before it is eliminated man must be re-created.

---

Toothache, when caused by acidity of the saliva acting on the exposed nerves, is promptly relieved by a strong solution of bi-carbonate of soda, used as a mouth wash and dentifrice.—*Exchange.*

Toothache is not caused by acidity of the saliva, and so the idea of using the solution named is nonsense. The acids of the mouth do not have their origin in the saliva.

---

Too Late for insertion in this number comes a communication from Dr. A. M. Dudley, informing delegates to the American Dental Association that he can secure for them the extremely low rates promised the Association if it went to California, with extension of time. If any desire to go, after the Niagara meeting, let them address Dr. Dudley, at Salem, Mass., without delay.

---

Dr. Dio Lewis, the Sanitarian, died May 20th. He was a very voluminous writer upon hygienic matters, and formulated more than one system which, if diligently followed, would preserve, he said, the body in perfect health for an indefinite time; yet Dr. Dio Lewis did not live out the allotted years of man.

---

It Costs $36 65 to govern a New Yorker; $7.40 to control an inhabitant of London; $7.35 of Berlin, and $5.40 a citizen of Paris. That is what the municipal government of each city costs, *per capita.* Are New Yorkers so comparatively refractory?

MANY DENTISTS who met Mr. C. A. Sykes, the genial agent of C. Ash & Sons, manufacturers of dental goods, London, when he was in this country, will be glad to know that he will return about the first of September. He comes in the interest of the Messrs. Ash.

---

DENTISTS WHO PROPOSE to attend the Niagara meeting should read the advertisement of the Prospect Park Hotel, in this number. It is an excellent house, is conveniently located, and its proprietor takes special pains to make his guests comfortable.

---

EVERY MEDICAL STUDENT is familiar with "Fehling's Test" for determining the presence of sugar in the urine. Prof. Fehling, of Stuttgart, who first proposed the reagent that goes by his name, has lately died at the age of seventy-two.

---

THE PRACTITIONER relates a case in which a lady was twice seriously poisoned by taking an egg in brandy. It was a personal idiosyncrasy, and neither the mother nor grandmother of the lady could eat eggs.

---

THE CHICAGO DENTAL SOCIETY lays out its work in advance. A programme has been issued containing the list of essays with the essayists for each meeting, completed to January, 1890.

---

DR. WILHELM HERBST, of Bremen, Germany, will be tendered a reception and dinner under the auspices of the First District Dental Society of New York, on Friday evening, July 2d.

---

DR. C. S. STOCKTON, of Newark, New Jersey, goes to Europe again for his summer vacation. He sails in July, and will spend the most of his time on the Continent.

---

DR. C. L. HUNGERFORD, of Kansas City, sails from New York for Europe about July 1st. He will spend the most of his time in Germany and France.

---

IT IS ALWAYS PROFITABLE to attend society meetings. One dentist has made $20,000 through attending the Minneapolis meeting last summer.

---

THE SOUTHERN DENTAL ASSOCIATION meets at Nashville, Tenn., Tuesday, July 27, and will remain in session four days.

---

THE MISSOURI STATE DENTAL SOCIETY will hold its annual meeting at Sweet Springs, commencing Tuesday, July 6th.

---

THE CALIFORNIA STATE DENTAL SOCIETY meets on Tuesday, July 20th, at San Francisco.

---

THERE ARE REPORTED 3,164 deaths from small-pox in Montreal last year.

